# Probiotic Potential of *Enterococcus lactis* GL3 Strain Isolated from Honeybee (*Apis mellifera* L.) Larvae: Insights into Its Antimicrobial Activity Against *Paenibacillus larvae*

**DOI:** 10.3390/vetsci12020165

**Published:** 2025-02-13

**Authors:** Manhong Ye, Yinhong Jiang, Qiannan Han, Xiaoyuan Li, Chuang Meng, Chao Ji, Feng Ji, Bin Zhou

**Affiliations:** 1 College of Bioscience and Biotechnology, Yangzhou University, Yangzhou 225009, China; mz120221741@stu.yzu.edu.cn (Y.J.); mz120231800@stu.yzu.edu.cn (Q.H.); 18326980175@163.com (X.L.); 2 Jiangsu Key Laboratory of Zoonosis, Yangzhou University, Yangzhou 225009, China; mengchuang@yzu.edu.cn; 3 Fubiao Biotech Co, Ltd., Huaian 211799, China; fbbee@126.com; 4 Institute of Animal Husbandry and Veterinary Medicine, Beijing Academy of Agriculture and Forestry Sciences, Beijing 100089, China; jifengji@sina.com; 5 College of Animal Science and Technology, Yangzhou University, Yangzhou 225009, China

**Keywords:** honeybee larvae, lactic acid bacteria, *Paenibacillus larvae*, *Enterococcus lactis*, aborycin

## Abstract

This study aimed to address the need for effective probiotics and antibacterial agents to combat American foulbrood disease in honeybees, caused by *Paenibacillus larvae*. In the context of declining honeybee populations due to pathogens, we isolated eight lactic acid bacteria (LAB) strains from honeybee larvae (*Apis mellifera* L.) and evaluated their probiotic potential and inhibitory effects against *P. larvae*. Methods included probiotic property assessments, such as acid and bile salt resistance, hydrophobicity, auto-aggregation, co-aggregation with *P. larvae*, antioxidant capacities, osmotolerance to 50% sucrose, and antibiotic susceptibility. Results indicated that the GL3 strain exhibited superior probiotic attributes and potent inhibitory effects on *P. larvae*. Whole-genome sequencing revealed GL3 to be an *Enterococcus lactis* strain with genetic features tailored to the honeybee larval gut environment. Pangenome analysis highlighted genetic diversity among *E. lactis* strains, while molecular docking analysis identified aborycin, a lasso peptide produced by GL3, as a promising inhibitor of bacterial cell wall synthesis. These findings suggested that GL3 was a promising probiotic candidate and antibacterial agent for honeybee health management, warranting further investigation into its in vivo efficacy and potential applications in beekeeping practices.

## Simple Summary

Honeybees are crucial for pollination, but their populations are declining due to diseases like American foulbrood, caused by the bacterium *Paenibacillus larvae*. This disease is lethal to honeybee larvae and can lead to colony collapse. To combat this, scientists are looking for probiotics, or beneficial bacteria, that can help honeybees fight off pathogens. In this study, researchers isolated eight strains of lactic acid bacteria from honeybee larvae and tested their probiotic properties and inhibitory effects against the growth of *P. larvae*. One strain, called GL3, stood out because it showed strong resistance to acid and bile salts, had good antioxidant properties, and could effectively inhibit the growth of *P. larvae*. Further analysis revealed that GL3 belonged to the species *Enterococcus lactis* and had genetic features that helped it survive and thrive in the honeybee larval gut. Additionally, GL3 could produce a compound called aborycin that had the potential to disrupt the cell wall of *P. larvae*, making it a promising antibacterial agent. These findings suggested that GL3 could be a valuable probiotic for honeybee health and may help reduce the impact of American foulbrood disease, benefiting honeybee populations and the ecosystems they support.

## 1. Introduction

Honeybees (*Apis mellifera* L.), crucial to pollination, ecological balance, and the production of honeybee-derived products, form the cornerstone of sustainable agriculture. However, the health of global honeybee colonies is threatened by a multitude of factors, including diseases, parasites, poor nutrition, pesticides, and the reduction in genetic diversity, all of which have important impacts on the well-being of these essential pollinators [1]. Among the various pathogens that pose a threat to honeybee health, the American foulbrood (AFB) pathogen, *Paenibacillus larvae*, is lethal to honeybee larvae and can cause massive colony collapse owing to the absence of larvae developing into adult honeybees. AFB disease is recognized as the most destructive bacterial disease exclusively affecting honeybee larvae [2], and a broad range of prevention and control measures have been devised to combat it [3].

Research shows that the honeybee microbiota is crucial for maintaining the host’s health by stabilizing the intestinal environment, defending against pathogens and parasites, aiding digestion and detoxification, and regulating immunity [4]. With the growing awareness of the importance of intestinal microflora in honeybee health, the incorporation of probiotics into beekeeping practices is increasingly recognized as a safe and promising alternative to conventional methods, such as antibiotics, for enhancing the overall health of honeybee colonies and thereby improving their overall disease resilience [5].

Among probiotic preparations, lactic acid bacteria (LAB) strains are the most widely used. Commensal LAB naturally inhabiting adult honeybees’ and larval guts are generally considered beneficial [6]. They play a pivotal role in maintaining host health and preventing diseases through competitive exclusion of intestinal pathogens and production of beneficial metabolites. They help enhance the hosts’ gut health, bolster their immunity, and increase their resistance to diseases [7]. In in vivo assays, supplementing honeybees with exogenous and endogenous LAB strains that exhibit anti-*P. larvae* activities has been demonstrated to be a promising approach to combating AFB infection. For instance, oral administration of nine LAB strains (two *Lactobacillus curvatus* strains, one *Enterococcus thailandicus* strain, and six *Weissella* strains, which were isolated from fermented feeds and food) to honeybee larvae and adult honeybees stimulated their innate immune response [8]. The addition of a mixture of 11 LAB strains (isolated from the honey crop) [9] and the *Lactobacillus kunkeei* strain (isolated from the gut of honeybees) [10,11] to the larval food of honeybees reduced the mortality percentage of larvae challenged with *P. larvae* spores. The administration of *Lactobacillus johnsonii* CRL1647 (isolated from the gut of honeybees) to honeybee colonies stimulated egg-laying, increased capped brood counts and honey storage, and enhanced colonization of bacteria belonging to *Lactobacillus* [12,13]. The supplementation of hives with a triple-strain lactobacilli consortium, including *Lactobacillus plantarum* ATCC 14917, *Lactobacillus rhamnosus* ATCC 55826, and *Lactobacillus kunkeei* BR-1 (isolated from a healthy honeybee hive) enhanced honeybee survival against *P. larvae* infection and beneficially modulated innate immunity and other host-response genes [14]. In a pilot study, administering a pollen suspension containing *Apilactobacillus kunkeei* V18 (isolated from honeybee guts) to AFB-infected honeybee colonies resulted in a reduction of *P. larvae* within two weeks, along with a significant increase in LAB populations in the digestive tracts of the honeybees [15]. These findings validated the probiotic attributes of the LAB strains and their positive contribution to honeybees’ fitness in the presence of *P. larvae* challenge. Consequently, probiotics are proposed as a viable approach for the sustainable management of AFB [16].

Concerning the source of probiotic strains, increasing evidence underscores the significance of utilizing host-specific strains [17,18]. Endogenous probiotic strains offer distinct advantages over exogenous ones, as they are inherently adapted to the host environment. This adaptation enhances their survival, colonization, and functionality within the gut, potentially minimizing adverse effects and enhancing the efficacy of probiotic interventions. Therefore, endogenous LAB strains, derived from the natural habitats and food sources of honeybees, such as their intestines, nectar, and pollen, are preferred for probiotic formulations aimed at controlling AFB disease. Current research on AFB control predominantly focuses on endogenous LAB strains isolated from the intestinal tracts of adult honeybees [19]. However, there is a notable lack of reports on the probiotic properties of larvae-derived LAB. The exploration of these naturally occurring probiotics within the microbiota of honeybee larvae remains a largely unexplored frontier.

In this study, we aimed to identify and characterize endogenous LAB strains with desirable probiotic potential from honeybee larvae. We investigated their antimicrobial activities against *P. larvae*, evaluated their probiotic potential, analyzed their antibiotic resistance profiles, and then selected a promising candidate. Through whole-genome sequencing, pangenome analysis, and molecular docking analysis, we aimed to gain insights into the genetic traits and potential probiotic properties of the candidate strain, contributing to the development of effective probiotics tailored for honeybees. We sought to uncover potential probiotic strains that could be further developed for targeted AFB interventions in honeybee larvae. This study underscores the value of exploring endogenous bacteria as a source of probiotics with unique and potentially superior benefits for honeybee larvae health, and highlights the importance of genetic analysis in understanding the probiotic properties of bacteria.

## 2. Materials and Methods

### 2.1. Isolation of LAB Strains from Honeybee Larvae

The honeybee larvae used in this experiment were obtained from three healthy honeybee colonies of *Apis mellifera* L. maintained in a local apiary in Yangzhou, Jiangsu Province, China. Four-day-old larvae were carefully removed from the comb. To isolate LAB strains from honeybee larvae, the entire bodies of twenty larvae were first rinsed three times using sterile PBS. These larvae were then homogenized to create a composite sample. After performing serial dilutions, the homogenate was spread onto De Man-Rogosa-Sharpe (MRS) agar plates, which were subsequently incubated for 24–48 h at 37 °C in an atmosphere enriched with 5% CO_2_. Single colonies were selected and further purified through at least two rounds of the streak-plate method. These purified colonies were then cultivated in MRS liquid media. The bacterial isolates underwent Gram staining and catalase reaction tests, and those found to be Gram-positive and catalase-negative were preserved in MRS broth containing 50% (*v*/*v*) glycerol at −20 °C for later molecular identification of the strains.

### 2.2. Agar Well Diffusion Assays

We evaluated the antimicrobial efficacy of the isolated LAB strains against the *P. larvae* YZU strain, the causative agent of AFB disease, which had been previously isolated and its whole genome sequenced by our team. In vitro inhibition assays were performed with slight modifications to the previously reported method [14]. Briefly, vegetative *P. larvae* YZU cells, cultivated in MYPGP medium, were suspended in 0.01 M PBS (pH 7.2) to an OD_600_ of 0.75 (1 × 10^8^ cells/mL) and evenly spread onto freshly prepared MYPGP agar plates (300 µL per 90-mm plate) using a sterile spreader. The isolated LAB strains were grown in MRS broth overnight, then centrifuged at 4500× *g* for 10 min, and washed twice with 0.01 M PBS. The resulting bacterial pellets were re-suspended in 0.01 M PBS to the concentration of 1 × 10^9^ cells/mL and filled into 6 mm wells on the agar plates (20 µL per well). After 24 h of incubation at 37 °C under a microaerobic condition (containing 5% CO_2_), the diameters of the inhibition zones were determined using a Vernier caliper, with measurements taken in millimeters by averaging the distances spanning from one edge of the clear zone, through the center of the well, to the opposite edge in three distinct orientations. Each LAB strain was tested in triplicate, with sterile 0.01 M PBS serving as the negative control.

### 2.3. Evaluation of In Vitro Probiotic Characterizations of Isolated LAB Strains

Preliminary assessments of the probiotic properties of isolated LAB strains, including acid resistance (exposure to pH 3.0 for 1 h and 3 h), 0.3% bile salt resistance (for 1 h and 3 h), cell-surface hydrophobicity, and auto-aggregation, were conducted following the details described previously [20]. The auto-aggregation ability is an important property of probiotics, enabling the bacteria to adhere to intestinal epithelial cells. In this study, we assessed the auto-aggregation capacity of isolated LAB strains following the previously outlined method [21]. Briefly, LAB strains were cultured at 37 °C for 18 h, subsequently collected by centrifugation at 8000× *g* at 4 °C for 10 min, washed twice using PBS (pH 7.2), suspended in PBS, and then adjusted to an OD_580_ of 0.5. The resulting bacteria suspensions were incubated at 37 °C, and the OD_580_ was measured at both 0 h and 24 h. The auto-aggregation percentage was calculated using the formula: auto-aggregation% = [1 − (OD_24h_/OD_0h_)] × 100, where OD_24h_ and OD_0h_ represent the absorbance values measured at the time-point of 0 h and 24 h, respectively. Co-aggregation tests of isolated LAB strains with *P. larvae* YZU were performed and calculated as outlined in the previous report [22]. The determination of the strain’s osmotic tolerance to 50% sucrose followed the previously published method [11]. The antioxidant activities of isolated LAB strains, specifically their scavenging abilities against hydroxyl radicals, DPPH radicals, superoxide anions, and ABTS^+^ radical cations, were evaluated using the methods described by Shi et al. [23]. The preparations of intact cells (ICs) and cell-free supernatant (CFS), as outlined by Zhang et al. [24], and cell extract (CE) as described by Ahire et al. [25], all adhered to previously published literature. Antibiotic susceptibility tests were performed according to EFSA guidance [26].

### 2.4. Whole-Genome Sequencing

The whole genomes of candidate strains were sequenced using the Illumina Novaseq 6000 platform at Tianjin Novogene Bioinformatic Technology Co., Ltd. (Tianjin, China). The details of whole-genome sequencing and assembly were described in our previous article [20]. The obtained draft genomic DNA sequences were annotated using Prokka [27], and the circular genomic map was constructed using the Proksee server (https://proksee.ca) (accessed on 18 August 2024) [28].

### 2.5. Species Confirmation

The species to which the GL3 strain belongs was confirmed by calculating both the average nucleotide identity (ANI) using the JSpeciesWS online service [29], and the average amino acid identity (AAI) using the EzAAI tool [30]. Comparative genomic analyses were performed using genome–genome comparisons in the TYGS server (https://tygs.dsmz.de) (accessed on 18 August 2024) [31].

### 2.6. Comparative Genomics Analyses

A total of 229 *E. lactis* strains were used for pangenome analysis (Supplemental File S1), which included the GL3 and GSL3 strains isolated in this study, one HL4 *E. lactis* strain previously isolated from 4-day-old honeybee larvae by our team, and 226 *E. lactis* strains available in the NCBI database (as of 19 August 2024). The GFF3 files for each genome, generated by Prokka, were used in the pangenome analysis performed using Roary v3.12.0 [32]. Roary results were visualized using an online server (http://jameshadfield.github.io/phandango/#/) (accessed on 26 August 2024). The carbohydrate-active enzymes (CAZymes)-encoding genes in the *E. lactis* genomes were annotated using dbCAN (v2) [33]. Genome annotations were further conducted using the web server RAST (Rapid Annotations using Subsystem Technology) [34] (https://rast.nmpdr.org) (accessed on 6 October 2024). Antimicrobial resistance genes (ARGs) were determined using the tool ABRicate (https://github.com/tseemann/abricate) (accessed on 6 October 2024). In silico multilocus sequence typing (MLST) analysis was performed to further explore the genetic relationships among *E. lactis* isolates. In the absence of an MLST typing scheme for the *Enterococcus lactis* species, we performed MLST analysis using the ISHAM consensus MLST scheme for the *Enterococcus faecium* species, which contained the following 7 unlinked genetic loci: *atpA*, *ddl*, *gdh*, *purK*, *gyd*, *pstS*, and *adk* (http://pubmlst.org/) (accessed on 18 October 2024). Each isolated strain was defined by a unique sequence type (ST) derived from the combination of alleles obtained at each locus.

### 2.7. Molecular Docking Analysis

In the present study, we hypothesized that the GL3 strain may interfere with the cell wall synthesis process of the bacterium *P. larvae* YZU, consequently inhibiting the growth of its vegetative forms. As is known, *mraY* encodes phospho-N-acetylmuramoyl-pentapeptide-transferase (EC 2.7.8.13), which is a key enzyme involved in peptidoglycan biosynthesis and is indispensable for bacterial growth [35]. This integral membrane protein, MraY, catalyzes the transfer of phospho-N-acetylmuramoyl-pentapeptide to undecaprenyl phosphate, thereby forming lipid I, a crucial intermediate in the formation of the bacterial cell wall. Several peptidonucleosidic antibiotics, such as liposidomycins, muraymycins, and caprazamycins, are known MraY inhibitors [36]. To investigate this hypothesis, we employed molecular docking analysis to evaluate the inhibitory effects of potential secondary metabolites produced by the GL3 strain on the MraY protein encoded in the *P. larvae* YZU genome. Genome-wide identification of gene clusters for secondary metabolite biosynthesis in the genome of the GL3 strain was performed using the antiSMASH web service (https://antismash.secondarymetabolites.org/) (accessed on 8 October 2024). Ultimately, we directed our attention to aborycin, a RiPP (ribosomally synthesized and post-translationally modified peptide), and performed a thorough analysis of its competitive binding interactions with 3 known inhibitors (liposidomycin A, muraymycin D2, and caprazamycin A) of MraY protein.

Molecular docking analysis was conducted using AutoDock Vina, with the MraY protein from *P. larvae* YZU as the rigid receptor, and aborycin, liposidomycin A, muraymycin D2, and caprazamycin A as flexible ligands. The detailed procedures for obtaining the protein structure and preparing ligands were previously described in Ref. [37]. In brief, the 3D structure of the MraY protein was predicted using the SWISS-MODEL server (accessed on 31 August 2024 via https://swissmodel.expasy.org/interactive). Subsequently, the protein receptor was prepared by adding polar hydrogens, calculating Gasteiger charges, and assigning AD4 types using AutoDock Tools (v1.5.7). The 3D structures of the ligands were retrieved from ChEBI in mol2 format (accessed on 31 August 2024 via https://www.ebi.ac.uk/chebi/) and then converted to .pdbqt using OpenBabel (v2.4.1). The docking results were visualized with Pymol software (v.3.0.4) and the Protein–Ligand Interaction Profiler web server (https://plip-tool.biotec.tu-dresden.de/plip-web/plip/index) (accessed on 8 October 2024).

### 2.8. Statistical Analysis

The statistical analysis was performed with SPSS Statistics V22.0 (IBM Corporation, Armonk, NY, USA) using the Kruskal–Wallis test, followed by the Mann–Whitney post hoc test for multiple comparisons. These analyses were applied to the following aspects: (1) the assessment of probiotic properties of the isolated LAB strains, including their resistance to acid and bile salts, hydrophobicity, auto-aggregation, and co-aggregation with *P. larvae* YZU, antioxidant capacities, and osmotolerance to 50% sucrose; (2) the characterization of genomic features of various *E. lactis* strains isolated from diverse hosts, specifically the number of CDSs and genome size, the distribution and abundance of annotated CAZyme-encoding genes, and the results of RAST annotation based on subsystems. A *p*-value less than 0.05 was considered statistically significant for all analyses.

## 3. Results

### 3.1. Probiotic Properties of Isolated LAB Strains

**Figure 1:**
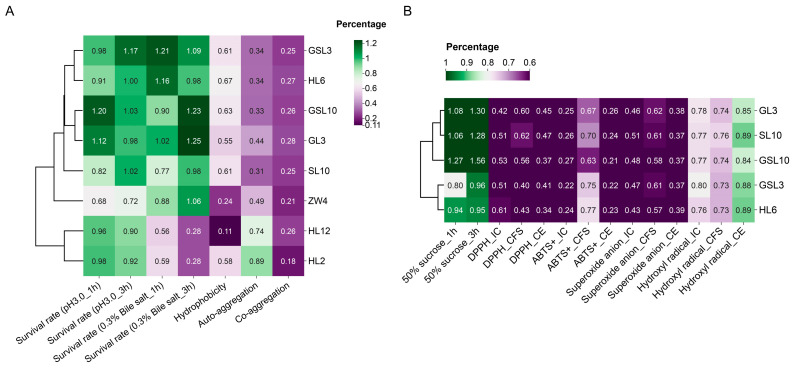
Performance of isolated LAB strains in terms of probiotic properties. IC, intact cell; CFS, cell-free supernatant; CE, cell extract. (**A**), probiotic properties of the isolated bacterial strains in terms of acid resistance, bile salt resistance, hydrophobicity, auto-aggregation, and co-aggregation with the *Paenibacillus larvae* YZU strain; (**B**), resistance to osmotic pressure induced by 50% sucrose and antioxidant properties.

We isolated eight LAB strains from 4-day-old honeybee larvae through pure culture on MRS agar plates. Results from the agar diffusion test revealed that strains GL3 and GSL10 produced the largest inhibition zones against the pathogenic strain *P. larvae* YZU, with diameters of 10.02 ± 0.10 mm and 10.10 ± 0.22 mm, respectively. Strains HL6 (8.98 ± 0.12 mm), GSL3 (8.91 ± 0.03 mm), and SL10 (9.14 ± 0.19 mm) produced moderately sized inhibition zones. Strains ZW4, HL2, and HL12 produced barely visible inhibition zones, suggesting no antagonistic activity against *P. larvae* YZU. With regard to the tested probiotic indicators, including acid and bile salt resistance, hydrophobicity, auto-aggregation, and co-aggregation with the bacterium *P. larvae* YZU, strains GSL3, HL6, GSL10, GL3, and SL10 outperformed the other three strains (Figure 1A). Based on these results, we excluded strains ZW4, HL12, and HL2 from our subsequent studies.

The results concerning the isolated strains’ antioxidant capacities and osmotolerance to 50% sucrose showed that strains GL3, SL10, and GSL10 exhibited better performance than the other two strains (GSL3 and HL6) (Figure 1B). The detailed results of the aforementioned tests were summarized in Supplementary Figure S1.

Results from the antibiotic susceptibility testing revealed that, among the nine antibiotics tested, five isolated LAB strains (GL3, GSL3, GSL10, SL10, and HL6) displayed identical sensitivity patterns for eight of the antibiotics, differing only in their resistance to streptomycin. Specifically, strains HL6, SL10, and GSL10 were sensitive to streptomycin, whereas strains GSL3 and GL3 exhibited resistance to it (Supplementary Table S1).

Our results demonstrated that the bacterial strain GL3 exhibited high tolerance to acid stress, maintaining a survival rate of over 98% after 3 h of exposure at pH 3.0 and demonstrating good tolerance to 0.3% bile salt. Additionally, GL3 displayed strong resistance to osmotic pressure induced by 50% sucrose, favorable antioxidant properties, and, most importantly, inhibitory effects on the growth of the pathogenic *P. larvae* YZU strain. These properties are crucial for the survival and colonization of probiotics within the host’s gut. The antagonistic activity against the growth of the pathogenic *P. larvae* YZU strain was an essential characteristic for GL3 to effectively combat this harmful pathogen and maintain gut health in the host. Considering these results comprehensively, we focused on the GL3 strain for subsequent whole-genome sequencing and further analyzed its potential as a probiotic strain tailored for honeybees.

### 3.2. General Genomic Features of the GL3 Strain

We conducted whole-genome sequencing of the GL3 strain to gain valuable insights into its genetic properties. The complete genome of the GL3 strain comprised one chromosome of 3.2 Mb with a GC content of 39.31% and harboring 2743 CDSs (coding DNA sequences) (Supplementary Figure S2). The species affiliation of the GL3 strain was confirmed by calculating both the average nucleotide identity (ANI) and the average amino acid identity (AAI), as well as through the phylogenetic analysis.

**Figure 2:**
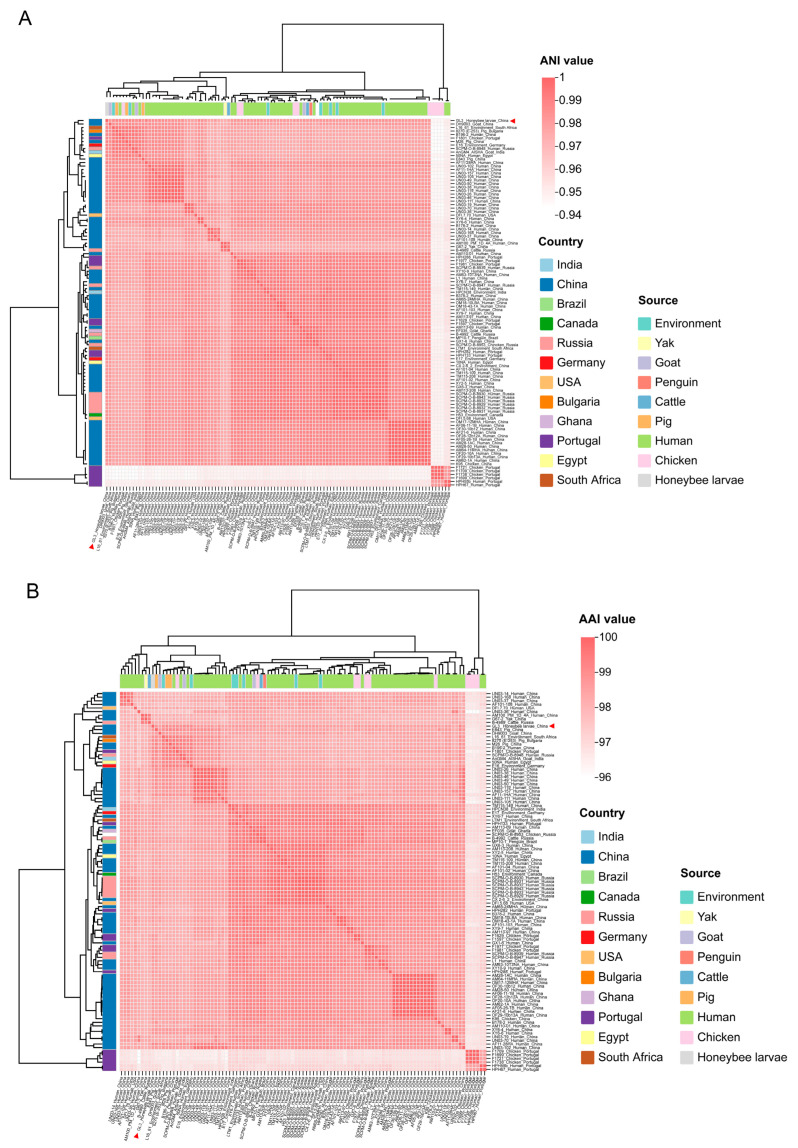

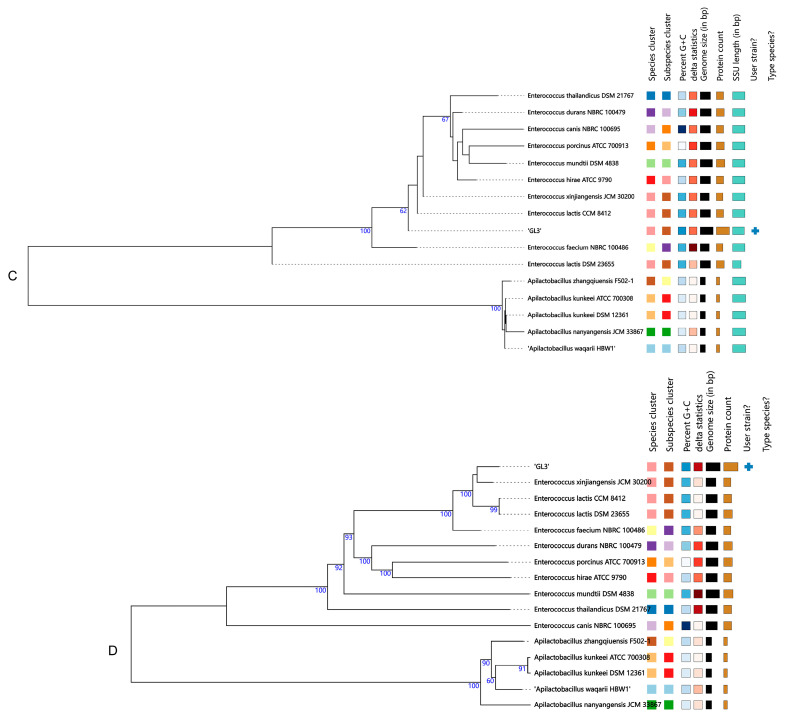
Species confirmation of the GL3 strain. (**A**,**B**), heatmap of ANI and AAI values, respectively, based on the sequences of the 105 *E. lactis* strains isolated from animals and the environment; (**C**), phylogenetic tree inferred with FastME 2.1.6.1 from GBDP (Genome-Based Distance Phylogeny) distances calculated from 16S rDNA gene sequences; (**D**), phylogenomic tree inferred with FastME 2.1.6.1 from whole-proteome-based GBDP distances. Branch values are GBDP pseudo-bootstrap support values > 60% from 100 replications, with an average branch support of 40.0% (**C**) and 90.8% (**D**), respectively. The branch lengths are scaled in terms of GBDP distance formula d5. The tree was rooted at the midpoint.

The ANI and AAI values across all 105 *Enterococcus lactis* strains exceeded 95% (Figure 2A,B), which was the threshold for species delimitation [38], confirming that the GL3 strain belonged to the species *E. lactis*. Additionally, strains isolated from the same source displayed higher ANI and AAI values compared to those from different sources, underscoring the source-specific nature of the *E. lactis* strains. Phylogenetic comparisons of the GL3 strain with representative complete genomes of other *E. lactis* strains, performed using the TYGS webserver, revealed that the GL3 strain was most closely related to *E. lactis* CCM 8412 (calculated based on 16S rDNA gene sequences) and *E. lactis* xinjiangensis JCM 30200 (calculated based on whole-proteome data) (Figure 2C,D). These findings further confirmed the taxonomic classification of GL3 as *E. lactis* with a high degree of confidence.

**Figure 3:**
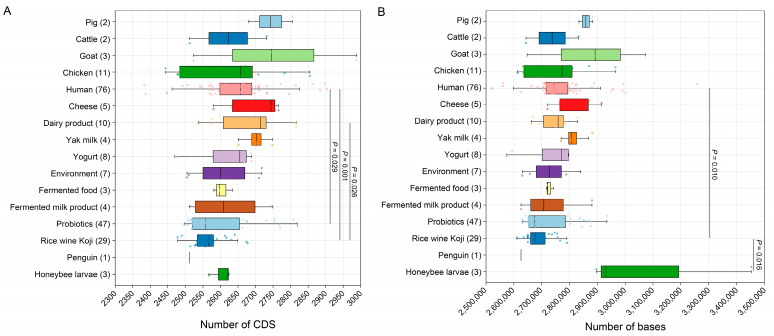
The number of CDSs and the genome size of 215 *E. lactis* strains isolated from different sources. The numbers in parentheses indicate the count of *E. lactis* strains. The Kruskal–Wallis test with Mann–Whitney post hoc test was used for comparisons. The only strain isolated from penguin was excluded from the statistical analysis. (**A**), the number of CDSs; (**B**), the number of bases in the genomes of different *E. lactis* strains.

Our results further revealed notable genetic diversity within the *E. lactis* species, as evidenced by variations in the CDS count and the genome size among strains from diverse origins (Figure 3). Significant differences in CDS number were primarily observed between *E. lactis* strains derived from humans and those isolated from probiotics or rice wine. Similarly, substantial variations in CDS number were noted between strains isolated from dairy products and those from rice wine. With regard to genome size, *E. lactis* strains isolated from rice wine Koji had significantly lower base count compared to strains derived from humans and honeybee larvae. It is worth noting that, while strains from other sources also displayed differences in CDS number and genomic size, these differences did not reach statistical significance due to the considerably smaller number of strains available for comparison, unlike those derived from human or probiotic sources.

### 3.3. Pangenome Analysis of E. lactis Strains

**Figure 4:**
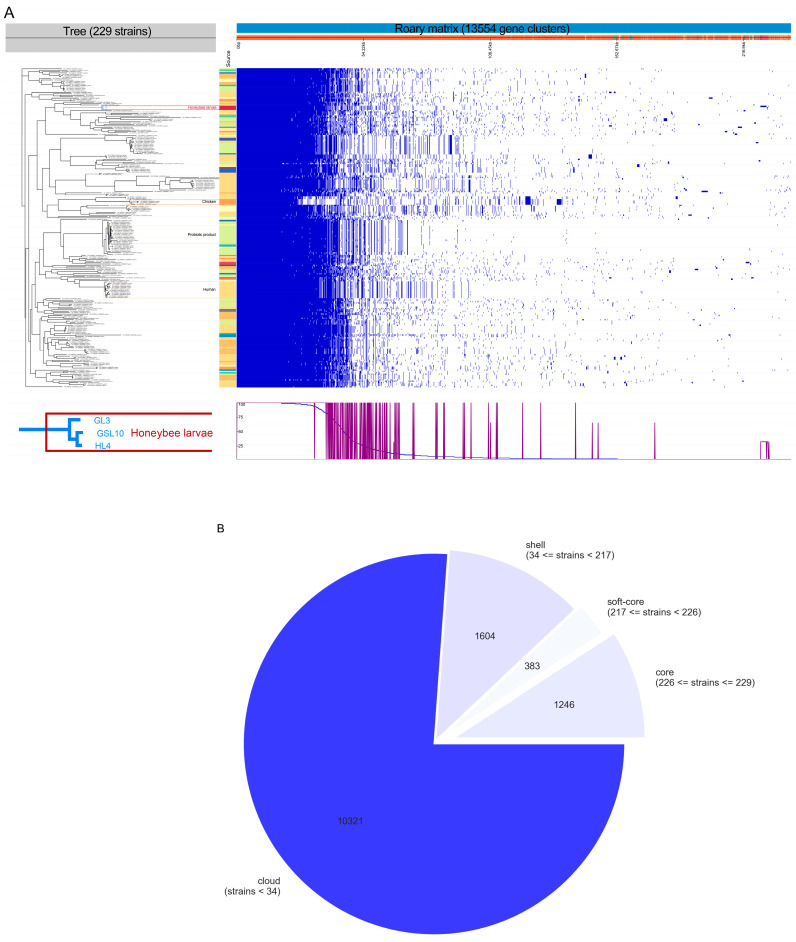

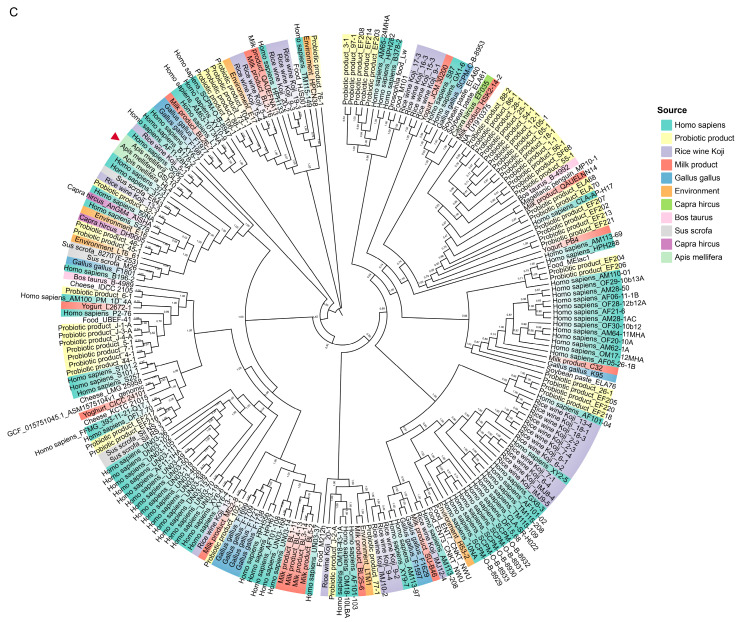
Results of the pangenome analysis using Roary. The genomic data of 229 *E. lactis* strains were used. Among them, the genomic sequences of 226 strains were retrieved from the NCBI database (as of 27 August 2024). GL3 and GSL10 are two *E. lactis* strains isolated from 4-day-old honeybee larvae in this study. HL4 is another *E. lactis* strain that was previously isolated from 4-day-old honeybee larvae, sequenced and assembled in our laboratory. (**A**), the matrix displaying the outcomes of the genomic comparisons among the 229 strains of *E. lactis* strains, where the blue and white stripes indicate the presence and absence of genes, respectively; (**B**), the pie chart showing the distribution of genes to the core, the soft core, the shell, and the cloud genes of *E. lactis*; (**C**), phylogenetic tree built on the gene presence/absence information produced by Roary.

The genetic diversity within *E. lactis* strains was reinforced by the pangenome analysis. Roary analysis revealed a pangenome integrated by 13,554 genes, with 1246 genes constituting the core genome and 12,308 genes belonging to the accessory genome (383 soft-core genes, 1604 shell genes, and 10,321 cloud genes) (Figure 4A,B). The accessory genes thus accounted for more than 90% of the pangenome (specifically, 76.1% as cloud genes), indicating substantial heterogeneity among these 229 *E. lactis* strains, and underscoring the open and dynamic nature of the *E. lactis* pangenome. The specific information of these 229 *E. lactis* strains used in Roary analysis are included in Supplementary File S1.

A phylogenetic tree was built to depict the relationships between all 229 *E. lactis* bacterial genomes, which also indicated a correlation between major clusters and their source or geographic origin (Figure 4C). Of note, the honeybee larvae-originated strains (GL3, GSL3, and HL4) formed a distinct clade from the other strains, suggesting the presence of gene families specific for honeybee larvae.

Exclusive genes identified among various *E. lactis* strains isolated from diverse sources are listed in Supplementary File S2. A total of 48 genes were identified exclusively in the genome of the GL3 strain. These genes encoded proteins that may assist the bacteria in efficiently utilizing nutrients within the gut (*dapD*, *fabG*, *dapB*, *yidA*, *maa*, and *glmS*), maintain intracellular stability (*cycA*, *glnQ*, and *crcB*), promote bacterial colonization within the intestinal environment (*ywqC*, *dapE*, and *nag3*), and enable sensing and responding to oxidative stresses within the gut (*walK* and *trxA*) (Table 1). Consequently, these genes may confer distinct advantages to the GL3 strain in the intestinal environment of the honeybee larvae. However, further experiments and research are needed to fully understand the specific roles of these proteins within the host’s gut.

### 3.4. Annotation and Functional Prediction of the GL3 Strain

In the present study, carbohydrate utilization-associated genes were annotated from the genome of GL3 using dbCAN2. A total of 2 auxiliary activity (AA) families, 4 carbohydrate esterase (CE) families, 9 carbohydrate-binding module (CBM) families, 44 glycoside hydrolase (GH) families, and 17 GT families were identified. Genes belonging to GHs and GTs were the most abundant gene families, highlighting their pivotal roles in carbohydrate metabolism within the 46 *E. lactis* strains. The number of CBM5-, GH154-, and GH18-encoding genes varied significantly among strains from different hosts. Specifically, strains from honeybee larvae had significantly more GH154 genes than those from chickens (*p* = 0.012) and humans (*p* < 0.001), as well as more CBM5 and GH18 genes than those from humans (both *p* = 0.010) (Supplementary Figure S3).

The same set of 45 genomes of *E. lactis* strains (Supplementary File S3) was further annotated using the RAST server. The RAST analysis revealed a total of 243 subsystems in the genome of the GL3 strain, with 23% subsystem coverage. Most of the genes were associated with protein metabolism (16.7%), carbohydrates (14.9%), and amino acids and derivatives (13.7%) (Supplementary Figure S4). When comparing *E. lactis* strains isolated from honeybee larvae to those of other sources, the larvae-derived strains exhibited significantly more genes associated with specific subsystems. Specifically, compared to human-derived strains, they had higher gene counts related to cell division and cell cycle (*p* = 0.007), fatty acids, lipids, isoprenoids (*p* = 0.003), and metabolisms of phosphorus (*p* = 0.008), potassium (*p* < 0.001), and RNA (*p* = 0.006). Compared to environment-derived strains, they had more genes linked to cofactors, vitamins, prosthetic groups, pigments (*p* = 0.014), and DNA metabolism (*p* = 0.042). The larval strains also contained a significantly higher number of genes involved in iron acquisition and metabolism when compared to those isolated from chicken (*p* = 0.036) and cattle (*p* = 0.006) (Supplementary Figure S5).

### 3.5. Detection of Antibiotic Resistance Genes (ARGs)

We further investigated the distribution of ARGs across the genomes of 121 bacterial strains originating from animals and the environment. Our results revealed that the distribution pattern of ARGs within the genome of *E. lactis* strains was related to their origin, with strains isolated from the same species and/or region exhibiting similar ARG profiles (Supplementary Figure S6). The majority of the *E. lactis* strains (95.9%) harbored resistance genes, namely, *aac*A-ENT1, *eat*(A), and *msr*(C), which encode aminoglycoside 6′-N-acetyltransferase, ABC-F type ribosomal protection protein Eat(A), and Msr(C), respectively.

### 3.6. In Silico Multilocus Sequence Typing (MLST) Analysis

A total of 50 distinct sequence types (STs) were identified among the 183 *E. lactis* strains analyzed (Supplementary Figure S7), underscoring the genetic heterogeneity amongst this species. ST60, ST94, and ST296 were the most frequently observed STs in strains isolated from human (18/57), human food (13/53), and probiotic products (18/47) respectively, indicating a potential association between specific STs (genotype) and their sources (ecological niche). Notably, ST2019, herein identified in three strains isolated from honeybee larvae, was not previously reported in any other strains, emphasizing the possibility of unique genetic profiles tailored to particular ecological niches. The gene network analysis further supported the notion of ecological specificity. The majority of strains isolated from humans, human foods, and probiotics clustered together and formed a closely connected gene network, while strains isolated from chickens, honeybee larvae, goats, penguin, and the environment constituted a distinct subset and were grouped separately from the main network. On the other hand, *E. lactis* strains isolated from humans and human-associated foods exhibited a scattered distribution across various MLST clades. Overall, our findings offered insights into the genetic diversity and potential ecological specificity of *E. lactis* strains. Detailed information on the 183 *E. lactis* strains, including their sources, isolation dates, geographic origin, and the ST results, is provided in Supplementary File S4.

### 3.7. Results of Molecular Docking Analysis

In our study, the *E. lactis* GL3 strain exhibited a large inhibition zone against *P. larvae* YZU. When the secondary metabolite biosynthesis gene clusters were analyzed using antiSMASH, it was revealed that GL3 harbored gene clusters predicted to encode aborycin, a ribosomally synthesized and post-translationally modified peptide (RiPP) (Supplementary Figure S8). We conducted molecular docking analysis to study the binding of aborycin to the enzyme MraY (phospho-MurNAc-pentapeptide translocase), a key enzyme for bacterial cell wall synthesis [35] encoded by *P. larvae* YZU, and compared it with the binding of three known bacterial MraY inhibitors, liposidomycin A, muraymycin D2, and caprazamycin A [36].

**Figure 5:**
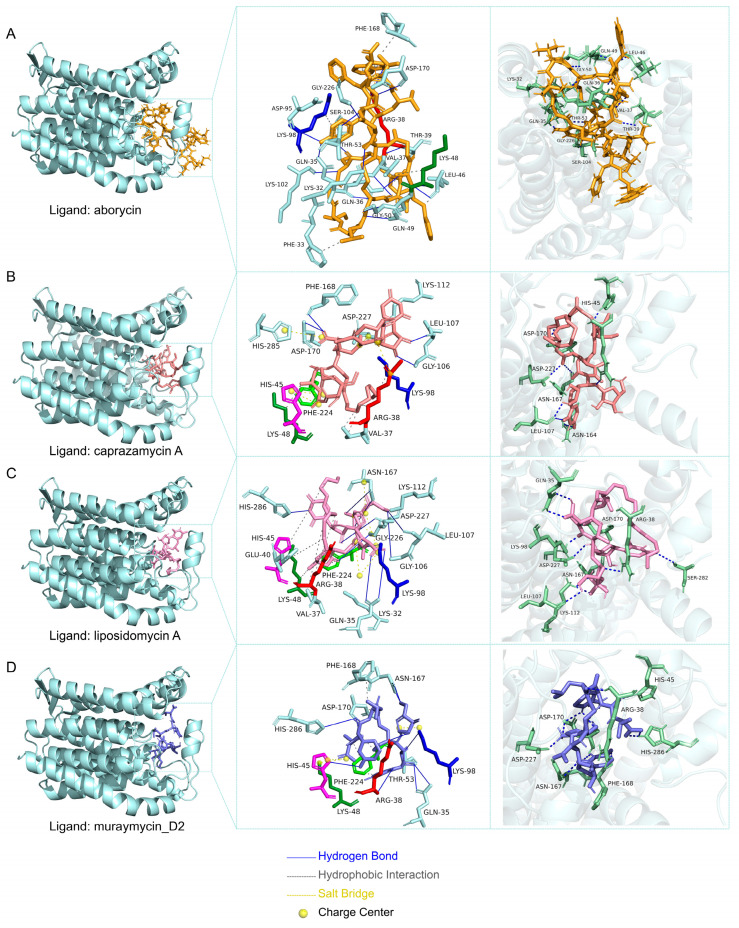
Binding interactions between the MraY protein, aborycin, and three other inhibitors. The protein structure of MraY (phospho-N-acetylmuramoyl-pentapeptide-transferase) encoded by the mraY gene in the genome of *Paenibacillus larvae* YZU was predicted with high confidence using the AlphaFold DB model (A0A1H5SM38_9BACL) corresponding to *Paenibacillus* sp UNC499MF. This model exhibited a Global Model Quality Estimation (GMQE) score of 0.94 and a sequence identity of 81.88%. The binding pocket in MraY was predicted by the DeepSite server (pocket centre: x, −9.11, y, −5.03, z, 14.46). (**A**–**D**) depict the close-up view of the in silico molecular docking results using AutoDock Vina showing the binding of MraY with aborycin, caprazamycin A, liposidomycin A, and muraymycin D2, respectively.

The results of molecular docking revealed that aborycin, as well as liposidomycin A, muraymycin D2, and caprazamycin A, all bound to the identical binding pocket of the MraY protein encoded by *P. larvae* YZU. This shared binding site suggested that these compounds targeted the same crucial site on the MraY enzyme. Additionally, aborycin demonstrated the highest affinity energy towards MraY, with a value of −8.6 kcal/mol, which surpassed the binding energies observed for liposidomycin A, muraymycin D2, and caprazamycin A with MraY (−8.1, −7.5, and −7.1 kcal/mol, respectively) (Figure 5). Aborycin’s superior binding affinity, compared to the three tested MraY inhibitors, indicated its potential to inhibit MraY activity more efficiently, thereby disrupting bacterial cell wall synthesis.

## 4. Discussion

In the present study, our findings not only confirmed the presence of LAB in honeybee larvae [22,53], but also demonstrated the inhibitory effects exerted by certain LAB strains against *P. larvae*, the causative agent of American foulbrood disease in beekeeping [19]. This underscores the importance of these beneficial bacteria in maintaining honeybee health.

The application of probiotics in apiculture as a viable alternative to antibiotics for disease management [54,55,56], as well as for the enhancement of honeybee health and productivity [57,58,59,60], has received considerable attention in recent years. In the current study, probiotic strains were selected based on specific biological functions critical for their efficacy in the hosts’ digestive tract, in addition to their antagonistic effects against the growth of the pathogenic *P. larvae* YZU strain. These probiotic properties included the gastrointestinal survival of the candidate strains, specifically, their tolerance to low pH and bile salts, their potential to adhere to the epithelial cells of the intestinal tract (as indicated by cell surface hydrophobicity), and their colonization potential along with the ability to prevent pathogen adhesion by creating a microenvironment around the pathogen (through auto-aggregation and co-aggregation) [61,62]. These properties, which enhanced their likelihood of proliferation and enabled them to exert the desired beneficial effects in the digestive tract of honeybee larvae, served as our primary criteria for selecting probiotic strains. Consequently, the LAB strains GL3, SL10, GSL10, GSL3, and HL6 were selected for further in vitro evaluations of their tolerance to 50% sucrose osmolality and antioxidant activities. When administering probiotics to honeybee colonies, sucrose syrup suspensions remain the most prevalent method due to their ease of application [63]. Honeybee colonies are typically fed a diet consisting of either 50% sucrose [64] or, in some cases, 50% glucose [65], supplemented with approximately 10^6^ cells/mL of probiotics. It was crucial to assess the tolerance of candidate strains to osmotic stress induced by 50% sucrose, in order to minimize rapid bacterial cell lysis and ensure their viability. In addition to these probiotic properties, the inclusion of probiotics with antioxidant activities could be an effective means of reducing oxidative stress in the intestines of the host [66]. Using this holistic strategy, we selected the GL3 strain, subsequently identified as *E. lactis* GL3, as our probiotic candidate. This GL3 strain not only addressed AFB management, but also demonstrated its reliability as a potential probiotic product. Future research will be directed towards validating the efficacy of GL3 in field trials and exploring its potential application in apiculture.

In addition to all of the aforementioned beneficial properties of GL3, its derivation from the natural host, namely, honeybee larvae, constitutes an advantage that cannot be overlooked. This host-specific origin is particularly favored in the selection and development of probiotics [67]. Compared to strains isolated from other sources, indigenous stains are more readily adaptable to the original host, spontaneously colonizing and proliferating, and thereby exerting their beneficial effects. In the last decade, several potential probiotic LAB strains have been isolated from the digestive tracts [10,11,13,54,60,68,69,70,71,72] and honey crop of honeybees [73]. These strains have demonstrated bacteriostatic activities against the pathogenic bacterium *P. larvae*, whereas, probiotic strains isolated from honeybee larvae have been relatively few [19,22]. The identification of *E. lactis* GL3 offers a promising potential for the development of probiotic preparations specifically targeted at honeybee larvae. This strain’s unique properties and host-specific origin make it a valuable candidate for further research.

Both the current study and previous reports consistently highlighted significant variations in CDS number and genome size among *E. lactis* strains derived from diverse sources [74,75]. These observations suggested that *E. lactis* strains may have undergone genetic diversification in the process of adapting to their unique ecological niches. To further explore this genetic diversity, we employed MLST, a robust method for characterizing bacterial populations based on the sequences of multiple housekeeping genes. The MLST results revealed a high degree of genetic diversity among *E. lactis* strains, which was consistent with the observed disparities in CDS number and genome size. This genetic diversity was particularly evident among strains isolated from distinct environments, further emphasizing the role of environmental factors in shaping the genetic makeup of *E. lactis* bacteria. The distribution pattern of STs further highlighted the existence of genetic clusters that correlated with specific environments. Strains from similar ecological niches tended to cluster together, indicating that they shared common genetic features that may be advantageous for survival and adaptation in those particular environments. This clustering not only highlights the adaptive potential of *E. lactis*, but also sets the stage for exploring the functional implications of this genetic diversity.

One such functional implication relates to the role of CAZymes in the assembly, breakdown, or modification of oligosaccharides, polysaccharides, and glycoconjugates [76]. Honeybees, which primarily rely on nectar as their main source of carbohydrates for energy production [77], present a unique ecological niche for *E. lactis*. After the initial three days of larval development, worker honeybee larvae transition from consuming royal jelly to a diet primarily consisting of pollen, honey, and nectar, known as “worker jelly” [78]. As they progress through larval development, their consumption of carbohydrates increases from 18% initially to 45% in the final two days [79]. While honeybees can readily digest sugars like maltose, trehalose, and melezitose, high doses of other sugars (such as galactose, mannose, lactose, raffinose, and other polysaccharides) can be toxic to them. Therefore, the bacterial community within honeybee larvae must possess a rich repertoire of sugar-metabolizing enzymes to efficiently utilize sugars and detoxify harmful substances. Our results revealed a significantly higher number of GH154- and GH18-encoding genes in three strains isolated from honeybee larvae, both of which belonging to the GH family that catalyze glycosidic bond hydrolysis, indicating an enhanced ability of these strains to hydrolyze specific glycosidic bonds. Along with the markedly increased abundance of CBM5-encoding genes, these enzymes collectively facilitate the degradation and utilization of the complex polysaccharides present in the honeybee larval diet. Our results suggested that *E. lactis* strains in honeybee larvae have undergone host-specific adaptations, possessing an abundant array of sugar-metabolizing enzymes to fulfill growth needs and detoxify harmful substances.

Moreover, compared to other LAB strains (such as *Enterococcus mundtii* NPL1379, *Lactobacillus paragasseri* NPL1369, and NPL1370), the genomes of *E. lactis* strains contained substantially more genes involved in the biosynthesis of cofactors, vitamins, and prosthetic groups [80]. The enrichment of genes related to the production of these essential nutrients in honeybee larvae-sourced *E. lactis* strains suggested increased self-sufficiency, conferring a survival advantage and facilitating colonization. Furthermore, their enhanced metabolism of iron, phosphorus, and potassium reflects efficient nutrient utilization, which contributes to their competitive advantage, resilience, and beneficial attributes within their ecological niche. Overall, these findings underscore the genetic and functional diversity of *E. lactis* strains originating from honeybee larvae and emphasize the importance of targeting this endogenous LAB population for the screening and development of potential probiotics, given their unique adaptations and beneficial traits tailored to the larval ecosystem.

In terms of antibiotic resistance properties, the *E. lactis* strains were found to have intrinsic resistance to aminoglycosides, macrolides, and pleuromutilin, conferred by the presence of *aac*A-ENT1, *eat*(A), and *msr*(C) genes, respectively. Our findings were consistent with a recent study that also detected *E. lactis* strains carrying resistance genes to aminoglycosides and macrolides [81]. These chromosomally encoded ARGs, located on the bacterial genomes rather than on mobile genetic elements, are less likely to disseminate resistance traits among bacterial populations. Their presence does not compromise the potential of these strains as probiotic candidates. In environments where antibiotics are present, this intrinsic resistance may even serve as an advantage, enhancing bacterial survival when antibiotic are co-administrated [82]. Notably, the relatively low abundance of resistance genes in the *E. lactis* strains isolated from honeybee larvae suggested that these strains have not been exposed to extensive selective pressure from antibiotics, highlighting the potential for using such strains in applications where minimal antibiotic resistance is desirable.

Aborycin, a lasso peptide exhibiting potential antimicrobial properties [83,84], was proposed to be a potent compound for inhibiting infection-causing bacteria, including *Staphylococcus aureus*, *Escherichia coli*, and *Pseudomonas aeruginosa* [85]. Our results revealed that GL3, which exhibited inhibitory activity against the pathogenic bacterium *P. larvae* YZU, harbored the gene encoding aborycin. Through molecular docking analysis, we demonstrated that aborycin specifically bound to the MraY protein, a crucial enzyme involved in bacterial cell wall synthesis in *P. larvae*. Remarkably, the binding site of aborycin on MraY overlapped with those of other known antibiotics that target this protein. This overlap suggested that aborycin may exert its antimicrobial effect through a similar mechanism of action, namely, by inhibiting the function of MraY and consequently disrupting the bacterial cell wall synthesis process. The strong binding affinity and specificity of aborycin for MraY provided a plausible explanation for the in vitro pathogenic antagonism exhibited by GL3 against *P. larvae* YZU. This finding not only underscores the potential of aborycin as a potent inhibitor of bacterial cell wall synthesis, but also highlights its promise as a specific antibacterial agent against *P. larvae* YZU. Future studies should focus on evaluating the in vitro and in vivo efficacy of aborycin against *P. larvae* YZU and exploring its pharmacokinetic properties, therefore, providing a deeper understanding of its antibacterial properties.

## 5. Conclusions

We isolated eight LAB strains from honeybee larvae, with the GL3 strain standing out for its inhibitory effects on the pathogenic *P. larvae* YZU and its probiotic potential. Whole-genome sequencing identified GL3 as *E. lactis* with unique genetic features tailored to the honeybee larval gut. Molecular docking analysis revealed aborycin, a lasso peptide potentially encoded by the GL3 strain, as a promising inhibitor of the MraY protein of *P. larvae* YZU. *E. lactis* GL3 is a potential probiotic candidate for honeybee health, meriting further in vivo investigation and exploration of its applications in beekeeping.

## Figures and Tables

**Table 1:** A subset of genes uniquely expressed in the genome of the *Enterococcus lactis* strain GL3 and their potential functions.

Gene	Annotation	Potential Functions
*dapD*	2,3,4,5-tetrahydropyridine-2,6-dicarboxylate N-succinyltransferase	Involved in metabolic pathways, particularly in the synthesis and degradation of amino acids [39,40], fatty acids [41], and sugars [42,43,44]. Help bacteria utilize specific nutrients in the gut of honeybee larvae to synthesize essential biomolecules, thereby gaining a competitive advantage in the intestinal environment.
*dapB*_2	4-hydroxy-tetrahydrodipicolinate reductase	
*fabG*_2	3-oxoacyl-[acyl-carrier-protein] reductase FabG	
*yidA*_4	Sugar phosphatase YidA	
*maa*	Maltose O-acetyltransferase	
*glmS*_3	Glutamine-fructose-6-phosphate aminotransferase	
*ywqC*_2	Putative capsular polysaccharide biosynthesis family protein YwqC	Involved in osmo-protection, biofilm formation [45], synthesis and degradation of bacterial cell wall polysaccharides or exopolysaccharides [46,47], potentially aiding in bacterial colonization within the intestinal environment.
*dapE*_2	Putative succinyl-diaminopimelate desuccinylase	
*nag3*	Beta-N-acetylglucosaminidase/beta-glucosidase	
*cycA*_2	D-serine/D-alanine/glycine transporter	Responsible for transporting various molecules and ions, potentially assisting bacteria in acquiring essential nutrients or excreting harmful substances within the intestinal environment [48,49,50]. Help bacteria obtain necessary nutrients or eliminate detrimental substances in the gut, thereby maintaining the stability of the intracellular environment.
*glnQ*_3	Glutamine transport ATP-binding protein GlnQ	
*crcB*	Putative fluoride ion transporter CrcB	
*walK*_2	Sensor histidine kinase WalK	Participate in bacterial signal transduction processes as a sensory protein, helping bacteria perceive and respond to changes in the intestinal environment [51]. Enable bacteria to rapidly adapt to micro-environmental changes within the host’s intestine.
*trxA*_3	Thioredoxin	An antioxidant protein with the ability to reduce reactive oxygen and reactive nitrogen species, protecting bacteria from the damage caused by oxidative stress [52].

## Data Availability

The whole genome sequences have been deposited at GenBank under the accession number of PRJNA1198306 (BioProject) and SAMN45835258 (BioSample) for the *Enterococcus lactis* GL3 strain and PRJNA1198847 (BioProject) and SAMN45853447 (BioSample) for the *Paenibacillus larvae* YZU strain, respectively.

## Supplementary Materials

The following supporting information can be downloaded at: https://www.mdpi.com/article/10.3390/vetsci12020165/s1. Figure S1: Performance of isolated LAB strains in terms of probiotic properties. IC, intact cell; CFS, cell-free supernatant; CE, cell extract. The Kruskal–Wallis test was used for comparisons. Figure S2: Circular map of the *Enterococcus lactis* GL3 strain genome visualized using the Proksee server. Starting from the outermost ring: Ring 1, Prokka annotation (+); Ring 2, Prokka annotation (−); Ring 3, Backbone (contigs); Ring 4, GC content; Ring 5, GC skew; Ring 6, mobileOG-db annotation. Figure S3: Distribution and abundance of annotated CAZyme-encoding genes in the genome of *E. lactis* strains isolated from different hosts. A total of 45 *E. lactis* strains were included the analysis (Supplementary File S3), primarily isolated from humans, animals, and the environment. The Kruskal–Wallis test, followed by the Mann–Whitney post hoc test, was conducted for comparisons. The heatmap represents the number of specific CAZymes. * and ** denote *p* < 0.05 and *p* < 0.01, respectively, highlighting significant differences observed among *E. lactis* strains isolated from different sources. Figure S4: General overview of biological subsystem distribution of the genes annotated using RAST-SEED server (https://rast.nmpdr.org) (accessed on 6 October 2024). Figure S5: Results of the RAST annotation based on subsystems. A total of 45 *E. lactis* strains were included the analysis, primarily isolated from humans, animals, and the environment. The Kruskal–Wallis test, followed by the Mann–Whitney post hoc test, was conducted for comparisons. The number in the bubble indicates the count of a specific subsystem category. * and ** denote *p* < 0.05 and *p* < 0.01, respectively, indicating significant differences among *E. lactis* strains isolated from various sources. Figure S6: Heatmap depicting the distribution of antibiotic resistance genes (ARGs) in the genomes of *E. lactis* strains isolated from various sources. The numbers in the heatmap represent the count of ARGs in the bacterial genomes. Figure S7: Results of multilocus sequence typing (MLST) analysis. (A), the pie chart reflecting the proportion of different sequence types (STs) among the 50 STs identified in 183 *E. lactis* strains. STs represented from one isolate each or two isolates are defined as other STs; (B), the circle packing graph reflecting the distribution of STs in *E. lactis* strains from different sources. The numbers in the circles represent the count of strains with a specific ST; (C), the goeBURST minimum spanning tree for all the 50 STs obtained from the combination of all allele types of the 7 MLST loci (*atpA*, *ddl*, *gdh*, *purK*, *gyd*, *pstS*, and *adk*) using the PHYLOViZ online server, indicating the genetic relationships between all the 183 isolates studied. Figure S8: Structure of aborycin. File S1: Information on the 229 *Enterococcus lactis* strains used for Pangenome analysis. File S2: Exclusive genes identified among various *Enterococcus lactis* strains isolated from diverse sources using Roary. File S3: Information on the 45 *Enterococcus lactis* strains used for CAZymes and RAST analyses. File S4: MLST results of 183 *Enterococcus lactis* strains. Table S1: Antibiotic susceptibility patterns of isolated strains.

## Abbreviations

The following abbreviations are used in this manuscript:

AFB	American Foulbrood
LAB	Lactic acid bacteria
ARGs	Antibiotic resistance genes
MLST	Multilocus sequence typing
