# Supplementary material for: Probiotic Potential of Enterococcus lactis GL3 Strain Isolated from Honeybee (Apis mellifera L.) Larvae: Insights into Its Antimicrobial Activity Against Paenibacillus larvae
Source: Vet Sci. 2025 Feb 13;12(2):165. doi: 10.3390/vetsci12020165 (PMC11861324; doi:10.3390/vetsci12020165)
Supplement: Supplementary file 1 [file vetsci-12-00165-s001.zip › supplemental files/Captions of all supplemental files.docx]

**Figure S1** Performance of isolated LAB strains in terms of probiotic properties

(S1A, resistance to acid and bile salts, hydrophobicity, auto-aggregation, and co-aggregation with *P*. *larvae* YZU; S1B, antioxidant capacities and osmotolerance to 50% sucrose; IC, intact cells; CFS, cell-free supernatant; CE, cell extracts. Kruskal-Wallis test was used for comparisons. No significant differences were observed.)

**Figure S2** Circular map of the *Enterococcus lactis* GL3 strain genome visualized using the Proksee server (Starting from the outermost ring: Ring 1, Prokka annotation (+); Ring 2, Prokka annotation (-); Ring 3, Backbone (contigs); Ring 4, GC content; Ring 5, GC skew; Ring 6, mobileOG-db annotation)

**Figure S3** Distribution and abundance of annotated CAZyme-encoding genes in the genome of *E*.

*lactis* strains isolated from different hosts. A total of 45 *E*. *lactis* strains were included the analysis

(supplementary File S3), primarily isolated from humans, animals, and the environment. The

Kruskal-Wallis test, followed by the Mann-Whitney post hoc test, was conducted for comparisons.

The heatmap represents the number of specific CAZymes. * and ** denote *p* < 0.05 and *p* < 0.01,

respectively, highlighting significant differences observed among *E*. *lactis* strains isolated from

different sources.

**Figure S4** General overview of biological subsystem distribution of the genes annotated using RAST-SEED server (https://rast. nmpdr. org) (accessed on 6 October 2024)

**Figure S5** Results of the RAST annotation based on subsystems.

A total of 45 *E*. *lactis* strains were included the analysis, primarily isolated from humans, animals, and the environment. The Kruskal-Wallis test, followed by the Mann-Whitney post hoc test, was conducted for comparisons. The number in the bubble indicates the count of a specific subsystem category. * and ** denote *p* < 0.05 and *p* < 0.01, respectively, indicating significant differences among *E*. *lactis* strains isolated from various sources.

**Figure S6** Heatmap depicting the distribution of antibiotic resistance genes (ARGs) in the genomes

of *E*. *lactis* strains isolated from various sources. The numbers in the heatmap represent the count of

ARGs in the bacterial genomes.

**Figure S7** Results of multilocus sequence typing (MLST) analysis.

(A), the pie chart reflecting the proportion of different sequence types (STs) among the 50 STs identified in 183 *E*. *lactis* strains. STs represented from one isolate each or 2 isolates are defined as other STs; (B), the circle packing graph reflecting the distribution of STs in *E*. *lactis* strains from different sources. The numbers in the circles represent the count of strains with specific ST; (C), the goeBURST minimum spanning tree for all the 50 STs obtained from the combination of all allele types of the 7 MLST loci (*atpA*, *ddl*, *gdh*, *purK*, *gyd*, *pstS*, and *adk*) using the PHYLOViZ online server, indicating the genetic relationships between all the 183 isolates studied.

**Figure S8** Structure of aborycin.

**File S1** Information on the 229 *Enterococcus lactis* strains used for Pangenome analysis

**File S2** Exclusive genes identified among various *Enterococcus* *lactis* strains isolated from diverse sources using Roary

**File S3** Information on the 45 *Enterococcus lactis* strains used for CAZymes and RAST analyses

**File S4** MLST results of 183 *Enterococcus lactis* strains

**Table S1** Antibiotic susceptibility patterns of isolated strains.
