# Supplementary material for: Probiotic Potential of Enterococcus lactis GL3 Strain Isolated from Honeybee (Apis mellifera L.) Larvae: Insights into Its Antimicrobial Activity Against Paenibacillus larvae
Source: Vet Sci. 2025 Feb 13;12(2):165. doi: 10.3390/vetsci12020165 (PMC11861324; doi:10.3390/vetsci12020165)
Supplement: Supplementary file 1 [file vetsci-12-00165-s001.zip › supplemental files/Table S1.docx]

| Group | Antibiotics | Cut off value (μg/mL) | HL6 | GSL3 | SL10 | GL3 | GSL10 |
| --- | --- | --- | --- | --- | --- | --- | --- |
| **Inhibitors of cell wall synthesis** | |  |  |  |  |  |  |
| Penicillins | Ampicillin | 2 | S | S | S | S | S |
| Glycopeptide | Vancomycin | 4 | S | S | S | S | S |
| **Inhibitors of protein synthesis** | |  |  |  |  |  |  |
| Single antibiotic | Chloramphenicol | 8 | R | R | R | R | R |
| Single antibiotic | Tetracycline | 4 | S | S | S | S | S |
| Lincosamides | Clindamycin | 1 | R | R | R | R | R |
| Macrolides | Erythromycin | 1 | R | R | R | R | R |
| Aminoglycosides | Kanamycin | 64 | R | R | R | R | R |
| Aminoglycosides | Streptomycin | 32 | S | R | S | R | S |
| Aminoglycosides | Gentamycin | 32 | S | S | S | S | S |

**Table S1** Antibiotic susceptibility patterns of isolated strains^*^

^*^ : Susceptibility is expressed as S, susceptible; R, resistant. Results were obtained from triplicates.
