# Supplementary figures and images for: Probiotic Potential of Enterococcus lactis GL3 Strain Isolated from Honeybee (Apis mellifera L.) Larvae: Insights into Its Antimicrobial Activity Against Paenibacillus larvae
Source: Vet Sci. 2025 Feb 13;12(2):165. doi: 10.3390/vetsci12020165 (PMC11861324; doi:10.3390/vetsci12020165)

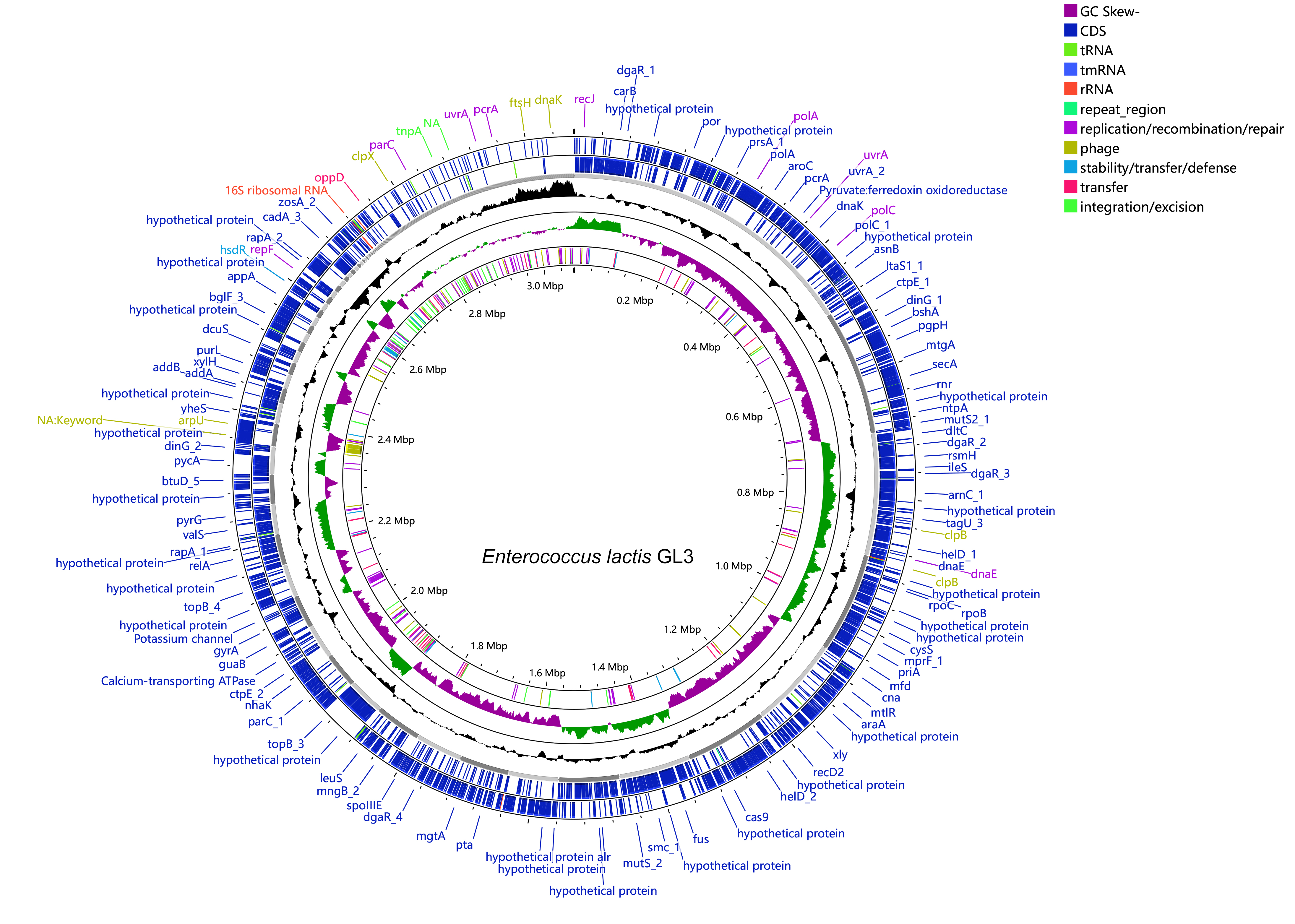

Supplement: Supplementary file 1 [file vetsci-12-00165-s001.zip › supplemental files/Supplemental Figure S2_Circular map of the GL3 strain genome.jpg]

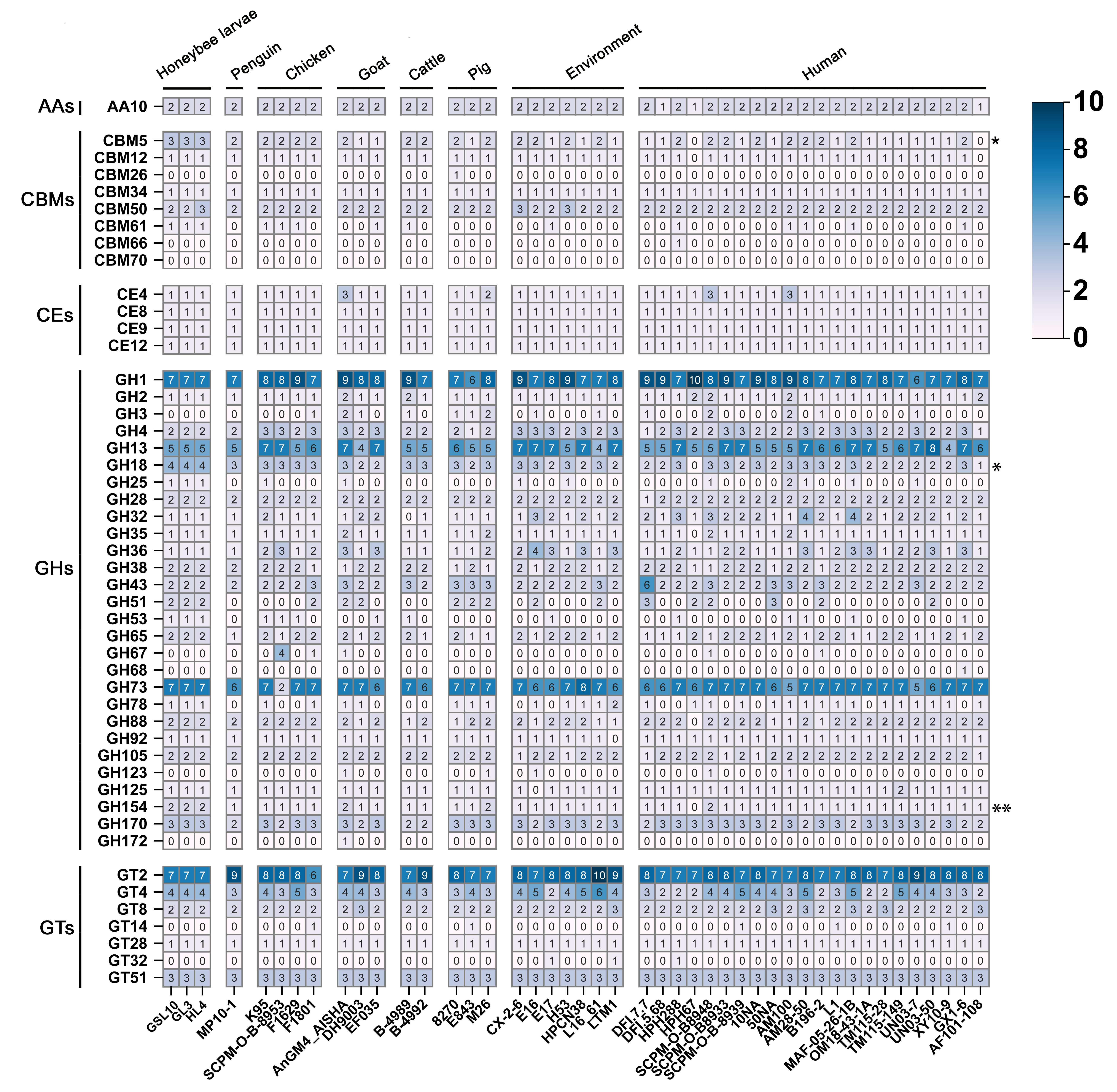

Supplement: Supplementary file 1 [file vetsci-12-00165-s001.zip › supplemental files/Supplemental Figure S3.jpg]

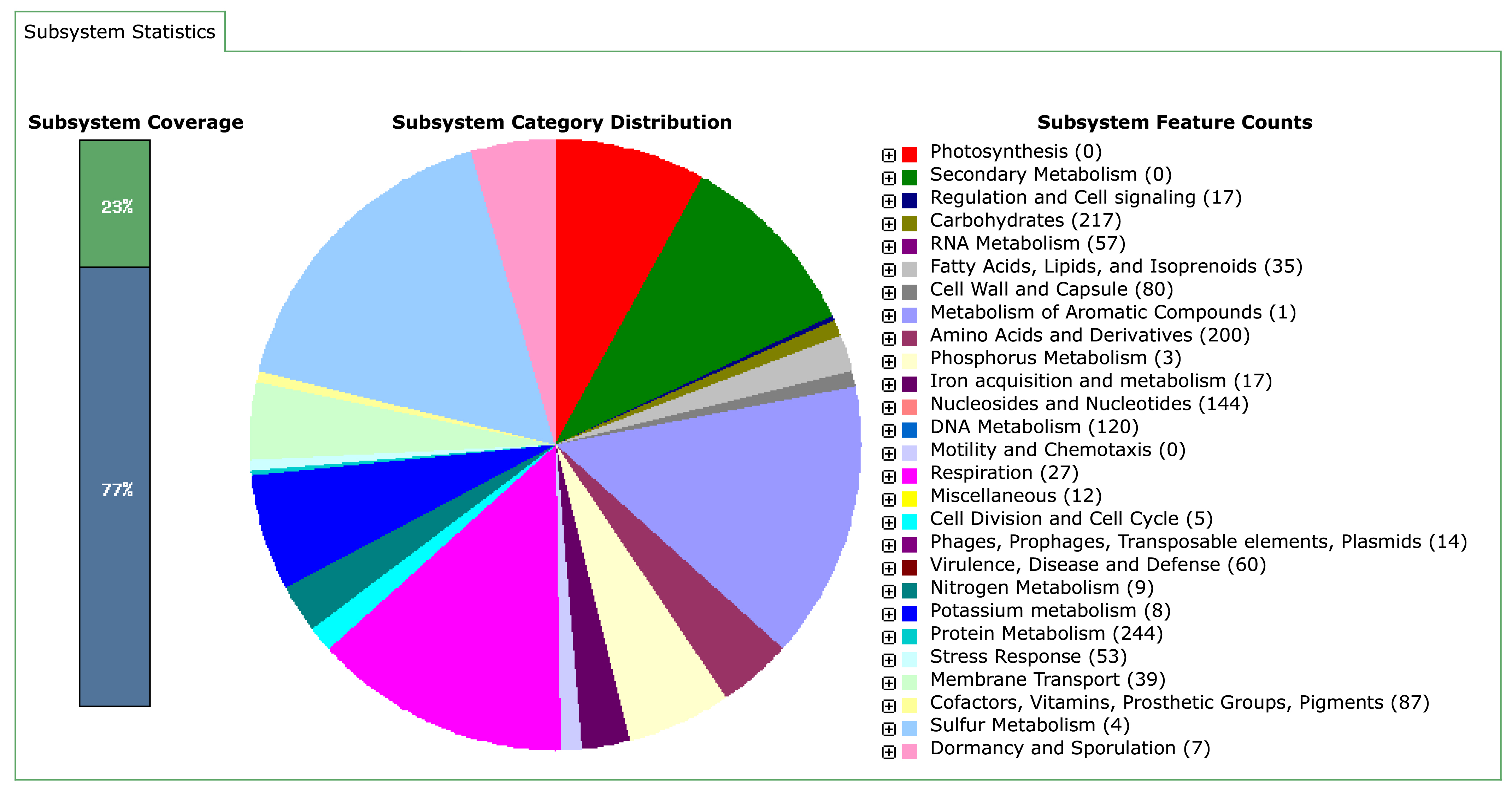

Supplement: Supplementary file 1 [file vetsci-12-00165-s001.zip › supplemental files/Supplemental Figure S4 RAST Seed Viewer results of GL3.jpg]

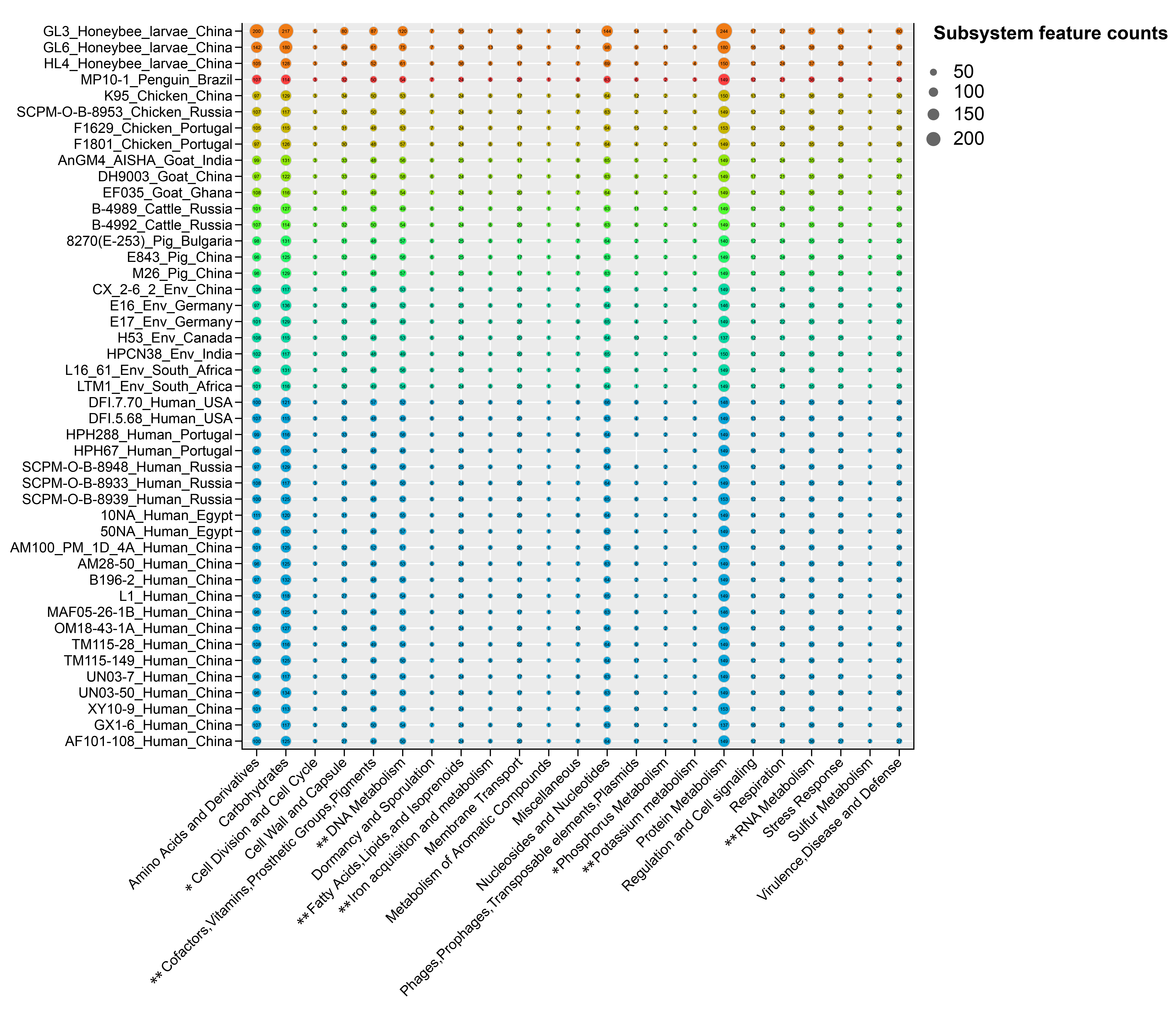

Supplement: Supplementary file 1 [file vetsci-12-00165-s001.zip › supplemental files/Supplemental Figure S5.jpg]

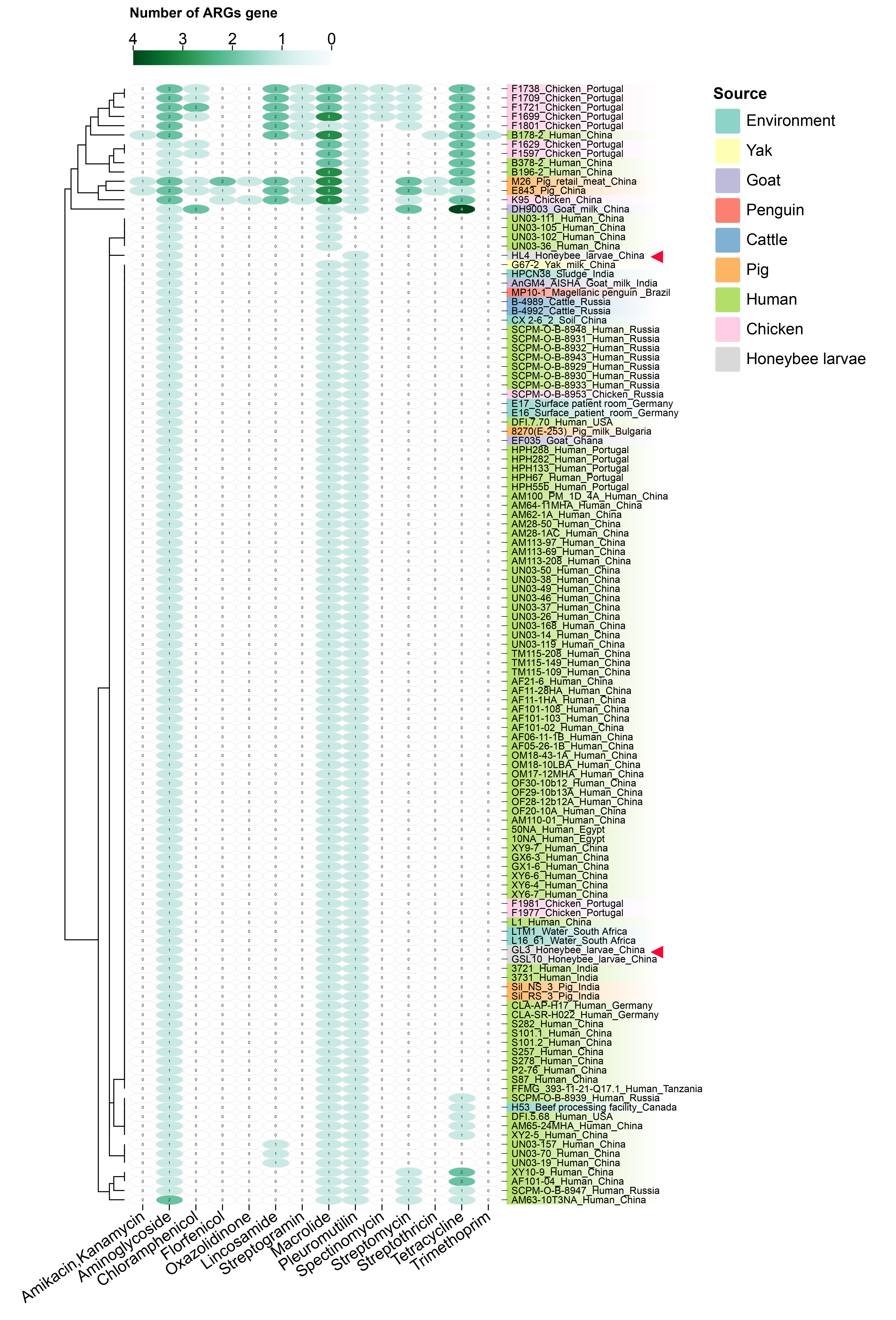

Supplement: Supplementary file 1 [file vetsci-12-00165-s001.zip › supplemental files/Supplemental Figure S6.jpg]

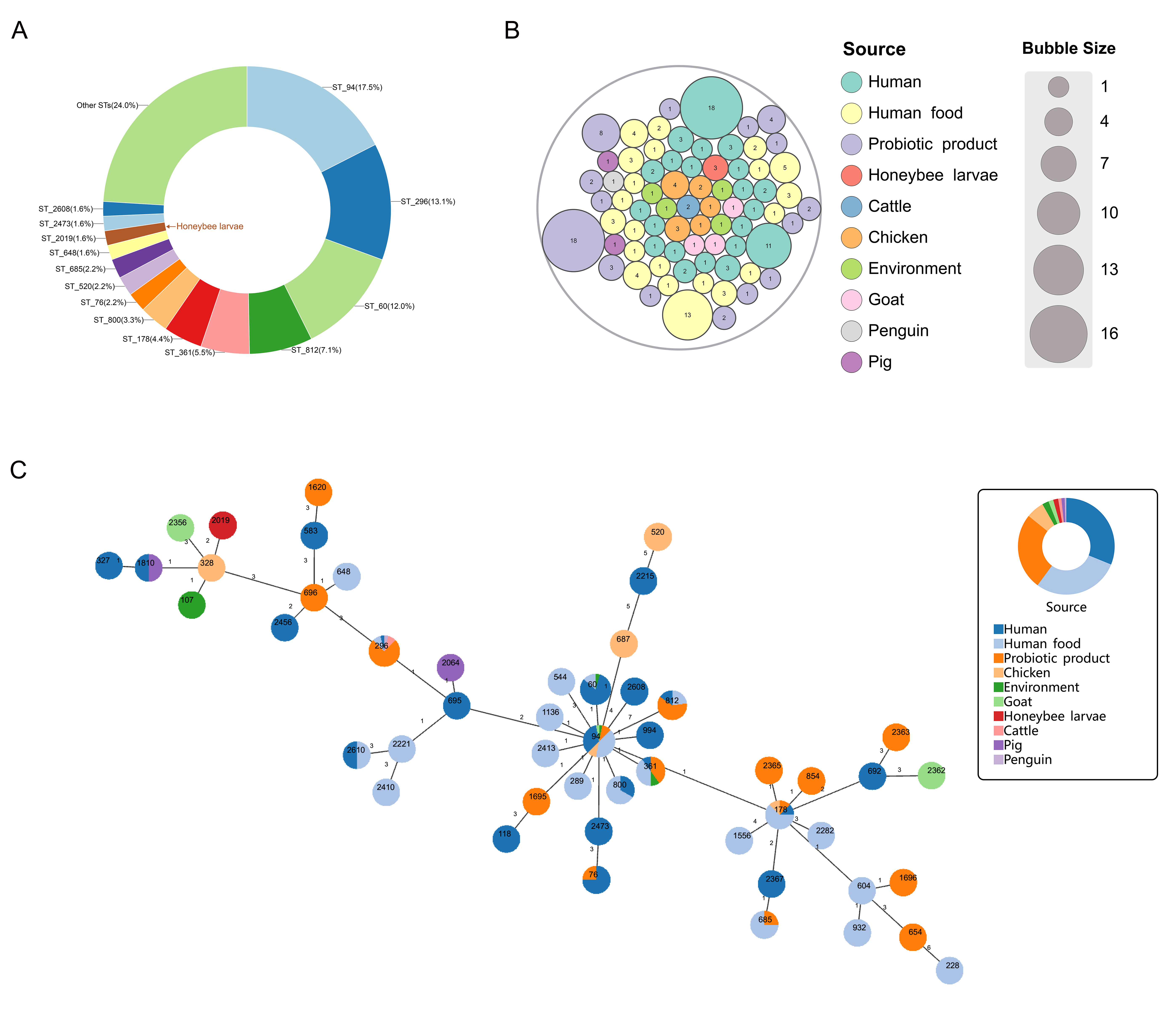

Supplement: Supplementary file 1 [file vetsci-12-00165-s001.zip › supplemental files/Supplemental Figure S7.jpg]

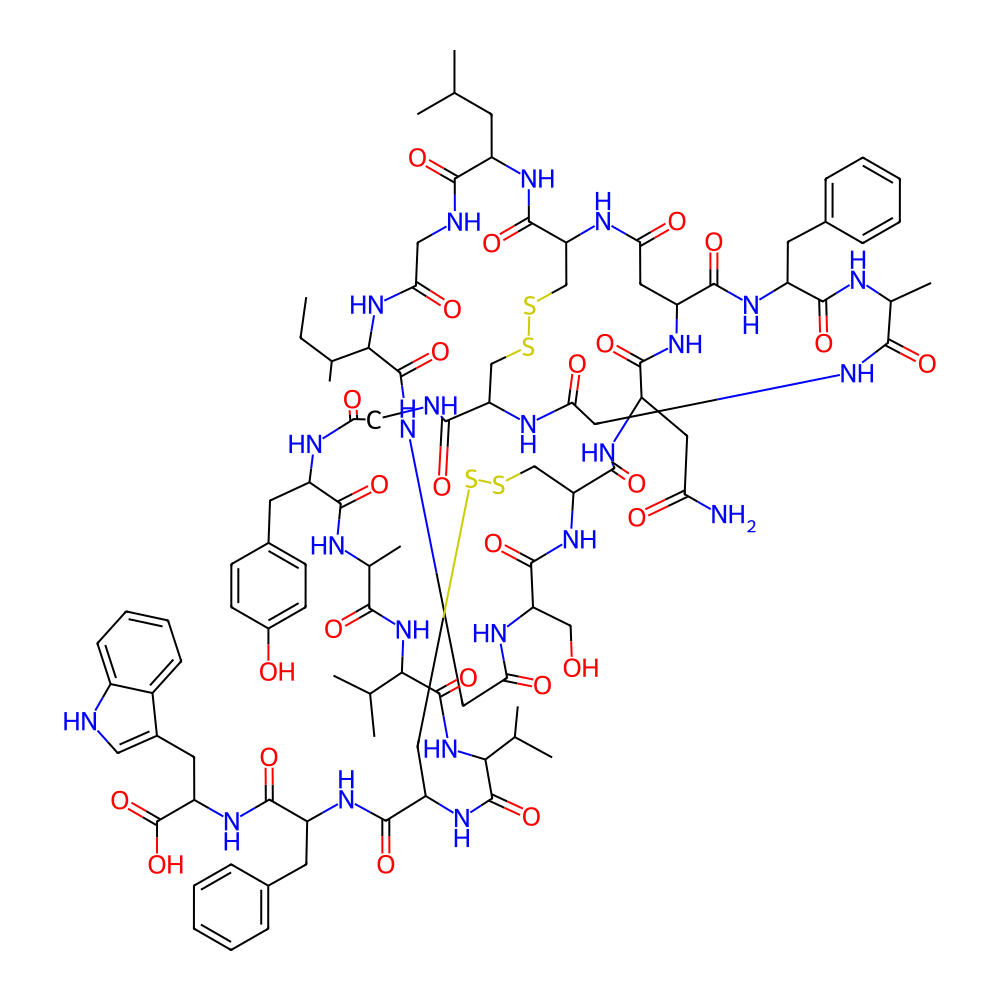

Supplement: Supplementary file 1 [file vetsci-12-00165-s001.zip › supplemental files/Supplemental Figure S8 Structure of aborycin.png]
